# Are Procoagulant Platelets an Emerging Therapeutic Target? A General Review with an Emphasis on Their Clinical Significance in Companion Animals

**DOI:** 10.3390/ijms26188776

**Published:** 2025-09-09

**Authors:** Meg Shaverdian, Austin Viall, Ronald H. L. Li

**Affiliations:** 1 Department of Pathology, Microbiology and Immunology, School of Veterinary Medicine, University of California, Davis, CA 95616, USA; mshaverdian@ucdavis.edu (M.S.); akviall@ucdavis.edu (A.V.); 2 Department of Clinical Science, College of Veterinary Medicine, North Carolina State University, Raleigh, NC 27606, USA

**Keywords:** aggregatory platelets, procoagulant platelets, hypertrophic cardiomyopathy, cardiogenic arterial thromboembolism

## Abstract

Platelets carry out their aggregatory and procoagulant roles in two distinct phenotypes. Aggregatory platelets initiate adhesion to the injured endothelium and extend the platelet plug, where procoagulant platelets accelerate thrombin formation and fibrinogen cleaving by exposing a procoagulant-rich outer membrane that facilitates coagulation factor assembly. Conventional anti-platelet therapies inhibit the aggregatory phenotype but fall short on restraining procoagulant platelets. Although procoagulant platelets are crucial for normal hemostasis, a shift toward excess procoagulant platelets is associated with human thrombotic disorders such as ischemic stroke. Although veterinary data is limited, recent studies show that feline and canine platelets display similar procoagulant phenotypes in response to potent agonists, suggesting that procoagulant platelets may play similar roles in the pathogenesis of thromboembolic disorders in veterinary species. Species-specific differences in platelet physiology and molecular structures, however, pose significant challenges. This review aims to (1) summarize cross-species evidence on the mechanisms driving procoagulant platelet formation, their defining features, and characteristics, (2) provide perspectives on procoagulant platelets as thrombotic biomarkers and outline the technical challenges of generating and detecting them in small animal medicine, and (3) summarize potential therapeutic targets and highlight priority research areas to advance the diagnosis and management of thromboembolic diseases in veterinary medicine.

## 1. Revisiting the Role of Platelets in Thrombus Formation

**Figure 1:**
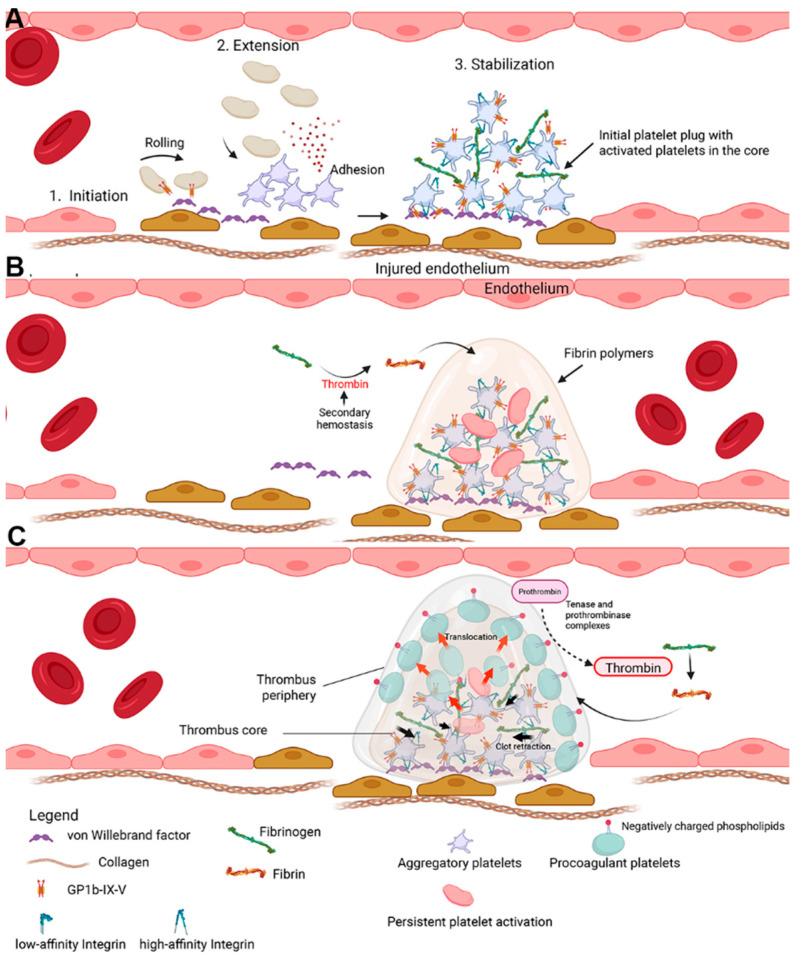
**Overview of thrombus formation and composition.** (**A**) Upon vascular injury, platelets undergo the three-stage model of platelet activation which involves platelet adhesion to subendothelial matrices and proteins such as collagen, extension (agonist release, shape change, and integrin (αIIbβ3) activation), and stabilization, which requires conformational changes in the integrin, αIIbβ3, and enabling platelets to aggregate by binding to ligands like fibrinogen. (**B**) The formed platelet aggregates during primary hemostasis enter its second phase during which the thrombus structure is further stabilized by the formation of fibrin polymers. Secondary hemostasis occurs on the membrane phospholipids that are exposed on the surface. Platelets are the primary phospholipid-exposing cells which provide a binding site for coagulation complexes assembly. These coagulation complexes, namely the tenase (factors VIIIa and IXa) and prothrombinase (factors Va and Xa), facilitate thrombin generation, which in turn cleaves fibrinogen to fibrin and leads to the formation of a stabilized fibrin clot. (**C**) Aggregatory platelets with their active αIIbβ3, form the majority of the thrombus core. As some platelets within the thrombus core are persistently activated due to exposure to agonists, they begin to adopt procoagulant platelet phenotypes characterized by high phosphatidylserine, a balloon shape, and loss of functional integrins. They are then translocated to the thrombus periphery (red arrows) due to clot refraction (black arrows). Highly exposed PS facilitates the binding of coagulation complexes to facilitate more thrombin generation and fibrin deposition to fortify the overall structure of the clot.

Platelets are the main effector cells in hemostasis, a complex physiologic process that prevents excessive bleeding due to vascular injuries and excessive clot formation that leads to thrombosis [1,2]. As the first responders to vascular injuries and hemorrhage, platelets undergo activation by the proposed three-stage model of activation, which includes initiation, extension, and stabilization (Figure 1A). In the initiation phase, subendothelial complexes, consisting of collagen and von Willebrand factors (vWF), are exposed secondary to endothelial injuries causing platelet adhesion and formation of a platelet monolayer. Under high shear stress, optimal platelet adhesion requires the binding of vWF to glycoprotein (GP) Ib subunit of GPIb–IX–IV receptor complex on the platelet surface. This binding, which is transient in nature, enables platelet tethering and rolling across the endothelium. As rolling platelets come into contact with collagen fibrils, collagen binds to glycoprotein VI (GPVI) on the platelet surface triggering platelet activation and marking the end of the initiation phase. Platelets enter the extension phase of activation when they are exposed to physiological agonists such as thrombin, thromboxane A_2_ (TxA_2_), adenosine diphosphate (ADP), and epinephrine. This results in further platelet activation and recruitment of nearby platelets and sets the stage for the initial platelet plug formation. During this time, platelets undergo important cellular events such as shape change, granule secretions, externalization of procoagulant phospholipids, and inside–out signaling. Inside–out signaling drives the conformational changes in the platelet integrin, α_IIb_β_3_, from a low affinity to high affinity state facilitating its binding to fibrinogen, vWF or fibronectin [1,2]. During the extension phase, the indirect adhesive interactions between neighboring platelets are mediated by the now activated integrins and their ligands. This facilitates platelet aggregation and initiates outside–in signaling cascade, which is characterized by additional granule secretion, irreversible activation, platelet spreading, and clot contraction caused by cytoskeleton reorganization to further narrow the gaps between platelets and to strengthen the overall platelet plug [1,2,3].

Although platelet activation and signaling is crucial for bleeding to stop, fine tuning of hemostasis is also warranted to prevent excessive bleeding or thrombosis. Platelets that partake in the initial platelet plug formation and cell-based model of coagulation are now classified as distinct subpopulations of platelets (aggregatory and procoagulant) [4,5]. Aggregatory platelets, historically recognized as activated platelets, express active high affinity α_IIb_β_3_ integrins for the initial platelet plug formation on injured endothelium through adhesive interactions with nearby platelets. This process is crucial in forming the thrombus core [4]. On the other hand, procoagulant platelets are known to provide a membrane surface suitable for binding of coagulation factors to augment thrombin generation [6]. The key difference in the formation of these subpopulations of platelets stems from multiple intrinsic and extrinsic factors influencing cellular mechanisms in response to agonists and the types of agonists [4].

The initial platelet plug is further strengthened by scaffolds of fibrin polymers assembled during secondary hemostasis, which is initiated when tissue factor (TF)-bearing cells, normally located outside of the vasculature, encounter activated factor VII (FVIIa) [5]. The newly formed TF-FVIIa complex initiates the cell-based model of coagulation and, through a series of enzymatic cascades, activates thrombin to cleave fibrinogen to fibrin (Figure 1B). In addition to clotting factors, platelets play an important role in the cell-based model of coagulation by providing a procoagulant membrane surface for the binding of clotting factors [5]. The asymmetry of the platelet membrane plays an important role in preventing excessive and spontaneous thrombosis. The enzyme, flippase, actively maintains the negatively charged phospholipids like phosphatidylserine (PS) and phosphoethanolamine in the inner leaflet of the phospholipid bilayer. In contrast, uncharged phospholipids are maintained on the outer leaflet by the enzyme floppase. Inside–out and outside–in signaling during platelet activation disturbs this asymmetry of phospholipid composition by externalizing PS to the outer leaflet to varying degrees [5]. Phosphatidylserine on the platelet surface and platelet-derived microvesicles facilitate direct interactions with coagulation factors by assembling the tenase (factors VIIIa and IXa) and prothrombinase (factors Va and Xa) complexes. This propagation phase of coagulation, which takes place on the platelet surface, generates large amounts of thrombin to cleave fibrinogen to fibrin monomers, resulting in the formation of polymerized fibrin clot (Figure 1) [5].

As platelets within the thrombus core are exposed to an extracellular milieu of potent agonists, a subset of platelets continues to undergo activation in a multi-step process [6]. This distinct subpopulation of platelets, known as procoagulant platelets, is translocated outward to the thrombus periphery by clot retraction creating a ring-like barrier around the thrombus core (Figure 1C) [6]. Procoagulant platelets, rich in PS on their surface, lead to the formation of a fibrin film around the thrombus, thereby strengthening the architecture of the clot. Procoagulant platelets, therefore, are crucial for overall thrombus stability [6]. However, based on the existing literature on human beings, platelets in individuals who have a higher tendency to adopt the procoagulant phenotype may be predisposed to thrombosis [7,8,9,10].

### Species Differences

Despite a shared function in hemostasis, platelets exhibit significant physiological differences across species in their size, count, sensitivity to agonists, and receptor expression. These key differences are essential to consider when studying platelets in companion animals and when extrapolating human research to veterinary medicine. While canine platelets resemble human platelets in terms of size, their membrane lipid composition is likely different considering that canine platelets have a larger range of thermotrophic membrane phase transition in response to cold temperature [11]. Additionally, canine platelets generate nearly twice the contractile forces during clot retraction compared to human platelets [12]. While studies directly comparing feline and human platelets are limited, feline platelets are known to aggregate spontaneously given their rapid aggregation response to ADP [13]. Receptor expression also differs considerably between species. While dogs and cats express the same key ADP receptors, P2Y_1_ and P2Y_12_, as humans, dogs and cats express significantly higher amounts of P2Y_1_ on their platelets compared to humans. Additionally, thrombin-induced activation in feline and canine platelets is mediated only via protease-activated receptor 1 (PAR1), instead of PARs 1 and 4 in human platelets [14]. Lastly, biochemical studies suggest that canine platelets do not express proteins present in human platelets such as platelet factor 4 and vascular endothelial growth factors [15].

## 2. History of Procoagulant Platelets

Prior to the identification of procoagulant platelets as a distinct subpopulation of activated platelets, platelet research centered around its role in supporting aggregation. The discovery of procoagulant properties of platelets, which possess similar membrane asymmetry to erythrocytes, was a major first step towards identifying procoagulant platelets [16,17,18,19]. The important role of platelets in propagating thrombin generation was first documented in human and canine patients with the congenital platelet disorder Scott syndrome, which is caused by variants in the TMEM16F gene responsible for encoding the enzyme, scramblase, which externalizes PS. Despite having normal aggregatory platelets, affected individuals often succumb to life-threatening hemorrhage due to the inability to externalize PS on their platelet surface [20,21]. The identification of ADP, collagen, and thrombin as the physiological agonists of platelets in 1960s was another step forward in studying procoagulant platelet function. A significant study by Bever et al. showed that co-stimulation of human platelets with thrombin and collagen resulted in substantial PS externalization compared to unstimulated platelets and platelets activated with either thrombin or collagen. As a direct result of PS externalization, prothrombinase (FVa and FXa) activity is significantly enhanced in platelets that are activated with thrombin and collagen [16]. A few years later, Alberio et al. demonstrated that platelets co-stimulated with thrombin and convulxin, a GPVI ligand, had high amounts of FV on their outer surface. This distinct population of platelets were later named Convulxin and Thrombin-induced-FV (COAT-FV) platelets” [22]. Various other studies introduced many names to better describe procoagulant platelets. For example, Kulkarni and Jackson wanted to emphasize the importance of sustained elevation of intracellular calcium in procoagulant platelets by naming them ‘Sustained Calcium-induced Platelet Morphology’ (SCIP) platelets [23]. Other names such as Collagen and thrombin combined stimulation (COATED) platelets and balloon-shaped platelets, were also given to describe procoagulant platelets [24,25]. Due to the lack of consensus among researchers, the term, procoagulant platelets, was proposed and universally accepted to better facilitate their clinical identifications and the understanding of their formation [4].

Although procoagulant platelets are crucial for hemostasis, there is mounting evidence suggesting that individuals with increased procoagulant platelets are at risk of cardiovascular-related morbidity and mortality such as ischemic stroke [7,8,9,10,26]. The procoagulant potential of platelets may, therefore, have diagnostic and prognostic value in predicting prothrombotic conditions in humans and animals. In addition, since currently used anti-platelet therapies only target the aggregatory function of platelets, procoagulant platelets may be a novel therapeutic target for thrombosis prevention. Despite recent advances in anti-platelet therapies for the treatment of cardiovascular-related thrombosis, recurrence rates remain high owing to the high interindividual variability in both humans and companion animals [27,28]. Thus, new therapeutic approaches that target procoagulant platelet formation might provide optimal thromboprophylaxis. It is important to note that most of our understanding of procoagulant platelets stems from human and murine platelet studies and the clinical and pathophysiologic relevance of procoagulant platelets remains unclear in companion animals.

In this review, we aimed to provide a comprehensive summary of the mechanisms of procoagulant platelet formation based on the existing literature; compare and contrast the cellular characteristics of procoagulant and aggregatory platelets; summarize and compare ways to detect procoagulant platelets as biomarkers in human and veterinary medicine and; identify the knowledge gap in veterinary medicine.

## 3. Mechanisms of Procoagulant Platelet Formation

Studies in both human and murine platelets have demonstrated that procoagulant platelets are generated through a multi-step process, but the precise mechanisms underlying their formation remain unclear. Even less is known about the species-specific differences in procoagulant platelet formation. Understanding the mechanisms underlying the generation of procoagulant platelets is an essential first step in uncovering novel therapeutics to optimize thromboprophylaxis in human and veterinary patients. Owing to the complexity and obvious species differences in platelet physiology, future research should focus on procoagulant platelets in veterinary patients with prothrombotic diseases.

### 3.1. Cytosolic Calcium Elevation via Both Store and Non-Store Operated Calcium Entry Mechanisms

**Figure 2:**
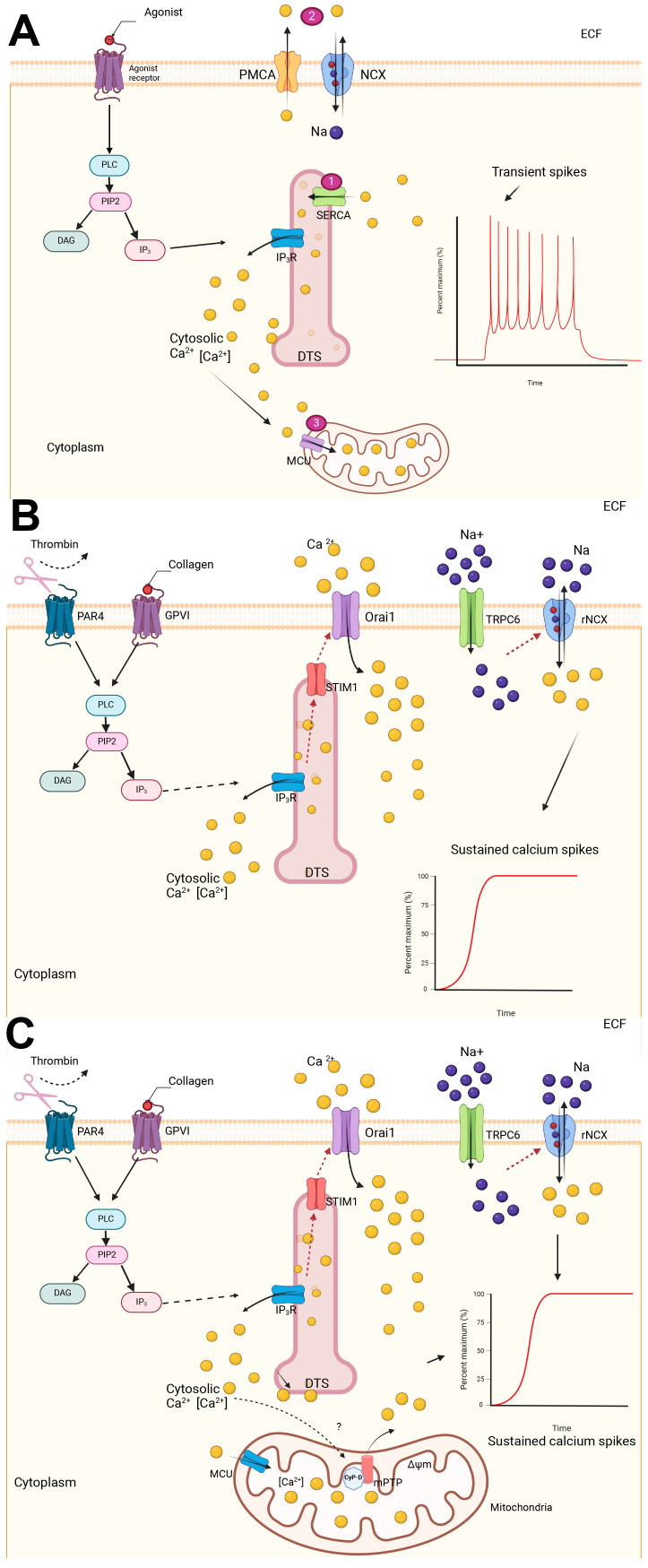
**Calcium signaling in procoagulant platelet formation.** (**A**) Transient intracellular calcium signaling. IP_3_ binding to its receptor, IP_3_R, on the DTS, results in calcium influx into the cytosol. Three main mechanisms are responsible for the maintenance of oscillatory intracellular calcium [Ca_i_] (1) [Ca_i_] triggers sarcoendoplasmic calcium ATPase (SERCA) to actively pump [Ca_i_] back into the DTS, (2) [Ca_i_] is transported out of the cytosol via plasma membrane calcium ATPase pump (PMCA) and NCX located on the plasma membrane. (3) Additional [Ca_i_] is stored within the mitochondria via MCU. This increase and decrease in [Ca_i_] creates an oscillatory [Ca_i_] pattern. (**B**) Sustained increase in [Ca_i_] is achieved via activation in response to platelet agonists such as thrombin and collagen. A sustained increase in [Ca_i_] is required for procoagulant platelet formation. This is achieved via calcium entry through plasma membrane in a process called SOCE. STIM1 is located on the DTS and is a regulator of ORAI1, the main channel involved in SOCE. STIM1 detects calcium depletion in the DTS and cytosol and signals ORAI1 to facilitate calcium entry into the cytosol from the extracellular space. TRPC6 allows sodium entry into the cytosol, which increases intracellular sodium concentration and reverses NCX to drive more calcium entry in exchange for intracellular sodium. (**C**) Electrochemical difference across the inner mitochondrial membrane drives calcium entry into the mitochondria via MCU. A marked increase in mitochondrial calcium concentration is sensed by cyclophilin D (CypD), which sets the threshold of of mitochondrial permeability transition pore (mPTP) opening. When the increase in calcium concentration reaches a certain threshold, mPTP allows vast amounts of calcium to enter the cytosol, creating a supramaximal calcium concentration. This is followed by activation of scramblase, TMEM16F, and the subsequent flipping of PS from the inner leaflet of the plasma membrane to the outer leaflet of the phospholipid bilayer. PAR4, protease-activated receptor 4; DAG, diacylglycerol; rNCX, reversed sodium-calciumsodium–calcium exchanger.

Intracellular calcium concentration [Ca_i_] plays a pivotal role in mediating signaling cascades essential for platelets to carry out their hemostatic functions [29]. In contrast to the intermittent or oscillatory elevation of [Ca_i_] required for aggregatory platelets, procoagulant platelets require a sustained and supramaximal increase in [Ca_i_] [30,31]. As platelets are continuously exposed to strong agonists within the thrombus core, the downstream signaling cascades activated by their corresponding receptors activate the enzyme, phospholipase C (PLC). Specifically, PLCγ2 operates downstream of GPVI following collagen engagement, while PLCβ is activated via PARs in response to thrombin. Additional input from ADP and thromboxane A_2_, acting through P2Y_12_, and TP receptors, further amplifies PLCβ activation. Once activated, PLC isoforms cleave phosphoinositide 4,5 bisphosphate to inositol 1,4,5 triphosphate and diacylglycerol. Inositol 1,4,5 triphosphate then binds to its receptors on the dense tubular system (DTS) leading to the release of calcium to the cytoplasm [29,31]. In homeostasis, several mechanisms are at play to maintain [Ca_i_] to oscillatory levels (Figure 2A) [29]. First, sarcoendoplasmic calcium ATPase 2b (SERCA2b), located in the DTS, actively pumps [Ca_i_] back into the DTS against an electrochemical gradient. Second, calcium efflux to the extracellular fluid is facilitated by sodium–calcium exchanger (NCX) and calcium ATPase pump (PMCA) on the plasma membrane to reduce [Ca_i_]. NCX, in its functional mode, pumps one calcium ion out in exchange of an extracellular sodium ion. Third, mitochondria buffers excess [Ca_i_] by storing calcium in the mitochondrial matrix via mitochondria calcium uniport (MCU). This dynamic interplay of calcium handling creates intermittent and transient spikes of [Ca_i_], which are essential in signaling pathways underpinning the functions of aggregatory platelets [29].

Persistent supramaximal [Ca_i_] due to continual activation is key to triggering intracellular signaling that drives platelets to become procoagulant. One way for platelets to achieve persistent supramaximal [Ca_i_] is through a process called store-operated calcium entry (SOCE) (Figure 2B) [29]. Store-operated calcium entry is a mechanism mediated by which the Stromal Interaction Molecule 1 (STIM1), located on the DTS, oligomerizes during low [Ca_i_] and stimulates calcium release-activated calcium channel protein 1, known as ORAI1, located on the plasma membrane to facilitate the calcium influx from the extracellular space to increase [Ca_i_] [29]. Other less eminent mechanisms of SOCE include the activation of transient receptor potential C (TRPC), which can be activated by calcium depletion or Gq-coupled protein receptor. Upon TRPC6 activation, sodium influx into the cytosol through TRPC6 reverses NCX causing calcium entry into the cytosol [32]. The important role of SOCE in procoagulant platelet formation has been investigated in mice and human platelets. Varga-Szabo et al. showed that mouse platelets lacking STIM1 have impaired calcium response and platelet activation upon agonist stimulation. In addition, STIM1 knockout mice are considerably less prone to thrombosis [33]. Similarly to STIM1-deficient mice, murine platelets lacking ORAI1 have significant reduction in calcium signaling, impaired agonist-induced platelet activation and thrombus stability in vitro [34]. The role of ORAI1 in the formation of procoagulant platelets is further confirmed by Abbasian et al., who showed that upon treatment of platelets with the ORAI1 blocker, Synta-66, a decrease in Fluo-4 fluorescence, a high affinity calcium indicator, was observed in procoagulant platelets. Strangely, signals of Fluo-5N fluorescence, which is sensitive to only very high levels of [Ca_i_], have little fluctuations in ORAI1-inhibited platelets. Thus, this shows that although ORAI1 is essential in the initial increase of [Ca_i_], other mechanisms are at play in the subsequent supramaximal calcium release [30].

### 3.2. Role of Mitochondria in Procoagulant Platelet Generation

As mentioned above, [Ca_i_] in platelets is regulated by the mitochondria. The electrochemical gradient across the inner mitochondrial membrane, also known as the mitochondrial membrane potential (ΔΨ_m_), favors calcium entry due to the negative ΔΨ_m_ in the matrix. Calcium crosses the outer and inner mitochondrial membranes via voltage-gated calcium channels and the MCU, respectively (Figure 2C) [35]. A substantial increase in mitochondrial calcium results in the opening of the mPTP, located on the inner mitochondrial membrane. mPTP is regulated by CypD, which sensitizes mPTP to calcium and is activated by reactive oxygen species. CypD, therefore, sets the threshold for mPTP opening [36]. When calcium accumulation within the mitochondria reaches a specific threshold, CypD facilitates mPTP opening, causing an calcium efflux from the mitochondria into the cytoplasm (Figure 2C) [36]. CypD-deficient mice and mice treated with CypD inhibitors have been shown to have lower numbers of procoagulant platelets due to increasing the threshold for mPTP opening. This indicates that CypD may play a key role in the commitment process of procoagulant formation [37,38]. The importance of CypD in necrosis likely explains the similarity in phenotypes between procoagulant and necrotic platelets. During necrosis, platelets experience reorganization of their cytoskeleton, mPTP opening, PS externalization, and ballooning. Thus, procoagulant platelets both mechanistically and morphologically resemble CypD-dependent necrosis [37]. Opening of mPTP and efflux of mitochondrial calcium to the cytosol creates sustained supramaximal [Ca_i_], activating scramblase, TMEM16F, and calpain, both required for externalizing PS (Figure 2C) [29].

## 4. Characteristics of Procoagulant Platelets

A better understanding of the cellular features of procoagulant platelets would allow researchers to identify procoagulant platelets in clinical patients and distinguish them from aggregatory platelets. The features of aggregatory and procoagulant platelets are summarized in Table 1.

### 4.1. Affinity for Integrin αIIbβ3

Aggregatory platelets are characterized by their activated α_IIb_β_3_ integrins, enabling them to aggregate with neighboring platelets due to the high expression of integrins on their pseudopodia [4]. In contrast to aggregatory platelets, procoagulant platelets have minimal ability to aggregate, as shown by Mattheij et al., who confirmed active proteolysis of α_IIb_β_3_ in procoagulant platelets. This lysis is mediated by calpain activation through active cleavage of the β_3_ unit of integrin in the presence of high calcium concentrations [38]. In addition, multiple human studies have shown that there is decreased binding to PAC-1, an antibody that recognizes the fibrinogen binding site epitope of α_IIb_β_3_, in procoagulant platelets compared to aggregatory platelets [39,40].

### 4.2. Phosphatidylserine Exposure

Externalization of the negatively charged PS on the external leaflet of the platelet membrane facilitates the binding of coagulation complexes, namely tenase and prothrombinase complexes, to augment thrombin generation [31]. However, not all platelets with externalized PS are considered procoagulant since apoptotic platelets also have externalized PS. By treating platelets from Bak and Bax knockout mice with the apoptosis inducer, ABT-737, Schoenwaelder et al. showed that mice lacking the ability to undergo caspase-dependent apoptosis do not express PS. On the other hand, when platelets from these knockout mice were stimulated with calcium or strong agonists like thrombin and collagen-related peptide, platelets regain the ability to externalize PS [41]. This highlights the mechanistic differences in PS flip between apoptotic and procoagulant platelets as the former is caspase-dependent and the latter is calcium-independent. For this reason, PS externalization alone cannot be used as a distinguishing feature. However, it can be used in conjunction with other markers to verify procoagulant platelets.

In addition to apoptosis and procoagulant platelet formation, the externalization of PS can also be found in a subpopulation of platelets with intact aggregatory functions. These platelets are found to express PS while maintaining high-affinity integrin α_IIb_β_3_ and lower [Ca_i_]. Their mechanism of formation and their role in hemostasis are unclear, but this subpopulation of platelets is likely generated via outside–in signaling due to platelet-to-platelet interactions by α_IIb_β_3_ [42]. The discovery of PS-positive aggregatory platelets suggests that the formation of procoagulant platelets is a highly dynamic process that is dependent on the platelet microenvironment. Furthermore, this indicates that PS alone is a non-specific marker of procoagulant platelets.

### 4.3. Morphology

A unique feature of procoagulant platelets is their transformation to balloon-like structures, which have been observed as part of a clot using electron microscopy [25]. Wedged between the balloon-shaped procoagulant platelets are fibrinogen and fibrin deposits that were cleaved by high concentrations of thrombin generated from bound coagulation complexes. In contrast, aggregatory platelets are discoid shaped, undergo shape change, and form extended pseudopodia upon activation [4,25,43]. The mechanism of this ballooning morphology was discovered by Agbani et al., who showed that calcium-activated chloride channel (CaCC) on the plasma membrane is essential for chloride and sodium influxes. Additional influx of chloride ions also occurs via the chloride/bicarbonate exchanger, which is dependent on carbonic anhydrase (CA) enzyme. The osmotic gradient generated by chloride and sodium influxes into the cytosol favors water movement into the cell via aquaporins 1 (AQP1s). A study using AQP1 knockout mice showed that it is essential in procoagulant spreading, budding of microvesicles, and in vivo thrombus formation. The absence of AQP1 expression, however, has no effect on platelet secretion, aggregation, and normal hemostasis [44]. Furthermore, when AQPs were inhibited by the carbonic anhydrase inhibitor, acetazolamide, ballooning of platelets, and thrombin generation decreased. These balloon-shaped platelets caused by water entry eventually give rise to platelet-derived procoagulant microvesicles, drastically increasing the surface area for coagulation complexes binding and thrombin generation [43].

### 4.4. Agonist Stimulation

Not all known platelet agonists are capable of inducing procoagulant phenotypes. Simultaneous stimulation of platelet PAR and GPVI with thrombin and collagen or convulxin, respectively, have been shown repeatedly to induce sustained cytosolic hypercalcemia and procoagulant platelet generation in vitro and ex vivo. Treatments of platelets with either a PAR or GPVI agonist can yield a lower ratio of procoagulant platelets. Other known platelet agonists such as ADP and TxA_2_ are unable to induce procoagulant phenotypes [7,38,41,45]. The authors’ recent experiences of studying procoagulant platelets in cats and dogs have shown that, similar to human and murine platelets, they exhibit procoagulant phenotypes in the presence of thrombin and collagen/convulxin [46].

## 5. Clinical Relevance

Globally, cardiovascular diseases (CVDs) remain the predominant contributor to death and disability, posing a major public health challenge. Hypercoagulability caused by platelets has been reported in various diseases such as ischemic stroke, peripheral artery disease, COVID-19, and heparin-induced thrombocytopenia (HIT) [26,47,48,49]. Although increased amounts of circulating procoagulant platelets are associated with disease severity in human patients, indicating a poor prognosis, the causative role of procoagulant platelets in disease pathogenesis remains unclear [47]. Nevertheless, given the association between procoagulant platelets and disease severity, procoagulant platelets may be utilized as a potential biomarker in the clinical risk assessment of thromboembolic diseases. In contrast to human medicine, veterinary medicine lacks systematic risk stratifications to identify animals at risk of thrombosis or hemorrhage. It is crucial to identify biomarkers such as procoagulant platelets to recognize at risk patients, who are predisposed to bleeding or thrombosis, so that appropriate preventatives can be administered to minimize morbidity and mortality.

### 5.1. Procoagulant Platelets in Cardiovascular Diseases

Procoagulant platelets have been studied extensively in humans with cardiovascular diseases such as coronary heart disease and ischemic stroke [7,8,9,10,26,50,51,52]. An increase in procoagulant platelets after ex vivo stimulation is documented in patients suffering from non-lacunar stroke and transient ischemic attack [9,26]. Pasalic et al. found that patients with coronary heart disease also have more procoagulant platelets than healthy patients upon treatment with thrombin and collagen ex vivo [7]. While increased procoagulant platelets are documented in humans with various cardiovascular diseases, only a couple of studies to date have identified the associations between thrombotic risk and procoagulant platelets [10,50,53]. Prodan et al. monitored 180 human patients with non-lacunar stroke and found that individuals who have the highest tendency to form procoagulant platelets (up to 70%) one year after the incident have the highest cumulative incidence of recurrent stroke [10]. Moreover, another study in 60 patients with symptomatic large-artery stenosis due to thrombosis illustrates the association between elevated procoagulant platelets with early stroke recurrence. Patients with documented 3-month recurrence of stroke were found to have significantly higher procoagulant platelets compared to controls [50].

While most of the focus of procoagulant platelets is on prothrombotic conditions, a modulated response to form procoagulant platelets may be associated with increased risk of bleeding. A pilot, single-center, study documented a difference in the distribution of procoagulant platelets between healthy individuals and people with spontaneous intracranial hemorrhage. When compared to race, sex and comorbidity-matched controls, the study found that patients with spontaneous intracerebral hemorrhage had persistently low levels of procoagulant platelets [52]. Another prospective study examined the association between mortality risk and procoagulant platelets and found that patients with the inability to mount a procoagulant platelet response (less than 27% positive platelets) have a 6-fold increase in mortality risk within 30 days after the initial intracerebral bleed [51].

### 5.2. Procoagulant Platelets in Thromboinflammation

Thromboinflammation is an important pathogenic process that mediates thrombosis and inflammation. Platelets are the key effector cells that bridge the gap between hemostasis, thrombosis, and inflammation. One mechanism in which procoagulant platelets partake in promoting inflammation is through the budding of platelet-derived microvesicles (PDMVs) [54]. Elevation of [Ca_i_] stimulates the activation of TMEM16F and calpain, which cleaves the membrane proteins such as actin-binding protein and disrupts the membrane resulting in PDMVs budding [55]. PDMVs, similar to their parent platelets, possess procoagulant features, providing a source of phospholipids for the assembly of coagulation complexes in the vasculature to further contribute to thrombin generation and coagulation. Increased thrombin formation can affect inflammation in multiple ways. First, thrombin can enhance intercellular adhesion molecule-1 (ICAM-1) expression, which mediates monocyte and neutrophil adhesion, and arrest on the endothelium and transcellular migration via diapedesis [56]. Second, thrombin can facilitate leukocytes movements towards the injury site by increasing cytokines production such as interleukin-8 (IL-8), which acts as chemoattractant guiding leukocytes throughout the tissues [57]. The proinflammatory cargos of PDMVs also enable its interactions with other immune cells such as neutrophils and monocytes. For instance, arachidonic acid donated from PDMVs to endothelial cells can upregulate ICAM-1 expression to further augment leukocyte arrest on endothelium and diapedesis [58]. Inflammatory chemokines such as C-C motif chemokine ligand 5 (CCL5) within PDMVs can enhance monocytes’ adhesion to the endothelium [59]. In addition, exposed P-selectin on the surface of PDMVs binds to P-selectin glycoprotein ligand-1 (PSGL-1) on monocytes and neutrophils resulting in the formation of leukocyte aggregates facilitating leukocyte activation [60,61]. Abnormally high levels of circulating PDMVs have been documented in pathological conditions including stroke, heparin-induced thrombocytopenia, venous thromboembolism, peripheral arterial disease, and myocardial infarction [62,63,64,65]. However, the caustic role of PDMVs in thrombosis remains unclear.

### 5.3. Procoagulant Platelets in Veterinary Medicine

Research on procoagulant platelets in veterinary species is limited. A study in dogs with lymphoma and solid tumors showed that ex vivo activation of platelets with thrombin and convulxin from dogs with solid tumors resulted in higher procoagulant platelets formation compared to healthy dogs [66]. However, this study only evaluated fibrinogen binding and none of the dogs were documented to have any thrombotic events. Parallel findings in human cancer patients underscore the translational relevance of procoagulant platelets. In individuals with colorectal cancer, both platelets and platelet-derived microparticles with PS are significantly elevated, promoting increased prothrombinase and tenase activity, accelerating clot formation and fibrin deposition [67]. More broadly, accumulating evidence highlights how tumor–platelet interactions drive a hypercoagulable state not only through elevated platelet counts but also altering platelet activation, function, and their procoagulant potential. Collectively, these findings suggest that tumor-driven priming of platelets toward a procoagulant phenotype represents a conserved and clinically relevant mechanism across species. Veterinary oncology may provide a valuable translational model for dissecting the mechanistic links between cancer, procoagulant platelet formation, and thrombosis [68,69].

In addition, to characterize procoagulant platelets formation in dogs with immune-mediated hemolytic anemia (IMHA), a highly prothrombotic disease, procoagulant platelet phenotype was characterized in an in vitro model of IMHA by treating platelets with extracellular heme in the presence or absence neutrophil extracellular traps (NETs). The authors found that hemin, a heme derivative, induces NET formation, which synergistically promotes procoagulant platelet formation. While hemin alone did not induce procoagulant platelet, it primed platelets to adopt a procoagulant phenotype in the presence of thrombin and GPVI agonists [70]. Additionally, NETs derived from hemin-treated neutrophils further enhanced procoagulant platelet formation, highlighting procoagulant platelets as a prominent player of immunothrombosis by linking neutrophil activation and thrombosis. This underscores their potential as both a therapeutic target and biomarker in veterinary medicine [70].

Decreased formation of procoagulant platelets leading to bleeding diathesis is a well-documented congenital coagulopathy in dogs. Better known as Canine Scott Syndrome, affected dogs are homozygous for the gene variant that encodes the protein, TMEM16F. This is likely to cause protein dysfunction, leading to significant decreases in PDMVs and externalized PS on platelets. Affected dogs have unpredictable bleeding diatheses such as prolonged spontaneous epistaxis, post-surgical hemorrhage and spontaneous hematomas [20]. Definitive diagnosis requires flow cytometry or genetic testing.

Although cats are prone to cardiogenic thromboembolism, the contribution of procoagulant platelets to the disease pathogenesis remains unclear. Cats with cardiomyopathies, especially those with severe hypertrophic cardiomyopathy (HCM) or transient myocardial thickening, have increased P-selectin expression and platelet priming in response to thrombin and arachidonic acid [71,72,73]. Cats with transient myocardial thickening due to smoke inhalation and wildfire burn injuries also had increased procoagulant PDMVs, suggesting a possible increase in procoagulant platelet formation and subsequent thrombin generation. The same study also found that sustained activation in response to arachidonic acid was independently associated with intracardiac thrombosis [73]. Although these studies have not specifically looked at procoagulant platelets, their findings suggest that procoagulant platelet activity might play a significant role in thrombus formation in cats with cardiomyopathies and other conditions associated with myocardial thickening. Further research is needed to elucidate the contribution of procoagulant platelets to cardiogenic thromboembolism in feline patients.

### 5.4. From Being Procoagulant to Exhausted: The Spectrum of Platelet Activation

While conditions such as inflammation or thrombosis favor procoagulant platelet formation, other contexts drive platelets toward a hyporesponsive or “exhausted” state. In high shear acquired von Willebrand syndrome, cleavage of ultra-large vWF multimers impairs platelet tethering and aggregation [74]. Mechanical circulatory support devices induce continuous shear stress and platelet surface receptor shedding, resulting in impaired signaling and “acquired platelet dysfunction” [75]. In advanced uremia, circulating toxins alter α_IIb_β_3_ activation and disrupt intracellular calcium handling, contributing to reduced aggregation and secretion [76]. Similarly, intensive anti-platelet therapy pharmacologically blunts platelet activation pathways, yielding functionally hyporesponsive platelets [77]. Collectively, these exhausted platelets exhibit attenuated granule release and poor procoagulant activity, a contrast to the hyperactive, prothrombotic phenotype seen in immunothrombosis.

## 6. Laboratory and Clinical Assessment of Procoagulant Platelets

### 6.1. Procoagulant Platelets as Potential Biomarkers

There is a critical need for a biomarker that can accurately predict not only the risks of thrombosis but also significant hemorrhage. Since procoagulant platelets play a crucial role in thrombin generation and fortification of clot structures, dysregulation in their formation and homeostasis can lead to either hemorrhage or thrombosis making them a potential biomarker for such risk assessments. The biggest limitation of measuring procoagulant platelets as viable biomarkers is the need for in vitro stimulation of washed or isolated platelets. This combined with the relatively short lifespan of platelets greatly limits the clinical utility of procoagulant platelets as biomarkers in clinical patients.

Recent advances in the study of procoagulant platelets have led to a better understanding of their morphology and characteristics, which has facilitated the development of more accessible and user-friendly assays to detect procoagulant platelets in the clinical and laboratory settings. Flow cytometry is the most utilized and least labor-intensive technique in studying procoagulant platelets given its ability to detect and quantify multiple surface markers simultaneously on a large number of cells. As mentioned above, many of the surface markers on procoagulant platelets can be detected using commercially available antibodies when studying human and murine platelets. Procoagulant platelets detection relies on the combination of markers to differentiate the phenotypes that are shared among apoptotic and aggregatory platelets. The International Society of Thrombosis and Haemostasias (ISTH) has published a set of guidelines recommending the simultaneous detection of 3 surface markers, P-selectin, PS, and GPIX/α_IIb_ integrin, to distinguish between procoagulant and apoptotic platelets in humans by flow cytometry [78]. These guidelines have some limitations when applied to the study of procoagulant platelets in veterinary species. While P-selectin and PS can be evaluated via flow cytometry, there are currently no antibodies like human PAC-1 to detect activated α_IIb_β_3_ integrin in veterinary species. Instead, an alternative marker needs to be utilized. Another limitation is that due to differences in platelet physiology, simultaneous detection of these markers on flow cytometry may not be feasible in all species. For instance, due to the sensitive nature of feline platelets to in vitro stimulation and exogenous calcium prolonged incubation of platelets with detectors predisposes platelets to spontaneously aggregate in the absence of agonists [46]. Given the limitations of studying procoagulant platelets in other species, a modified approach to the ISTH guidelines is required.

In addition to the aforementioned markers, there are other surrogates that can be utilized to characterize procoagulant platelets such as Δψm, the cell death marker, 4-[N-(S-glutathionylacetyl)amino]phenylarsonous acid (GSAO), fibrinogen binding, platelet-derived microvesicles, measurement of Ca_i_ and factor X activation. In this section, we will first summarize the ISTH recommended markers, followed by other ancillary markers (Table 2). Procoagulant assessment in veterinary medicine is discussed below.

### 6.2. P-Selectin

P-selectin is a transmembrane glycoprotein located on the alpha granule membrane. During platelet activation, P-selectin is translocated to the platelet surface when alpha granule membrane fuses with the platelet membrane upon degranulation [2,46]. Although there are other markers that can assess platelet activation, P-selectin is the most used platelet activation marker in veterinary medicine [46,73,79]. Studies using the apoptosis inducer, ABT-737, have demonstrated minimal P-selectin expression in platelets undergoing apoptosis, suggesting that platelets do not undergo alpha granule secretion during apoptosis [7,45,80]. Thus, P-selectin is one of the recommended markers to differentiate between procoagulant and apoptotic platelets. While P-selectin is a sensitive marker of platelet activation in dogs and cats, species differences in platelet sensitivity to agonists, ambient temperature, and shear stress must be taken into consideration. It is in the authors’ experience that prolonged exposure of canine and feline platelets to thrombin or calcium can lead to shedding or internalization of P-selectin, causing insufficient detection of P-selectin [73].

### 6.3. Phosphatidylserine Detection

Annexin V and lactadherin are commonly used to detect externalized PS via flow cytometry. Annexin V is a protein that binds to negatively charged phospholipids in a calcium-dependent manner. On the other hand, lactadherin is a glycoprotein, which binds to PS in a calcium-independent process [81]. Some studies have reported lactadherin being more sensitive in detecting PS compared to annexin V. A human study found that compared to annexin V, lactadherin can detect low levels of PS during the early stages of platelet activation. Annexin V and lactadherin were, nevertheless, both equally sensitive at detecting higher amounts of PS when platelets were fully activated [82]. A disadvantage of annexin V is its calcium dependency, which requires the addition of exogenous calcium and may, in turn, promote calcium influx and undesirable in vitro activation and, in some cases, apoptosis [78]. This is especially true in feline platelets when an abrupt increase in extracellular calcium may lead to clot formation preventing annexin V binding. We therefore recommend the addition of calcium to isolated platelets in aliquots over a period of 30 min to 1 h to avoid in vitro activation [46]. Overall, the ISTH guidelines recommend the use of annexin V over lactadherin as the binding of lactadherin on platelet integrins may lower its sensitivity to detect procoagulant platelets [78]. An additional advantage of annexin V is that it is more affordable and is commercially available in many conjugated formats, including direct conjugation with fluorophores.

### 6.4. Decreased α_IIb_β_3_ Integrin Affinity

As mentioned above, procoagulant platelets have proteolyzed α_IIb_β_3_. The magnitude of integrin proteolysis can be measured using species specific antibodies such as PAC-1 and JON/A to target the fibrinogen binding sites on human and murine platelets, respectively. PAC-1 has been used in human studies to differentiate between aggregatory and procoagulant platelets as procoagulant platelets are expected to show a significant reduction in PAC-1 positive cells [39,40].

### 6.5. Alternative Markers for Studying Procoagulant Platelets

#### 6.5.1. Inner Mitochondrial Membrane Potential (Δψm)

Depolarization of Δψm is a prerequisite to procoagulant platelet generation, which can be measured using cell-permeable fluorescent cationic dyes like tetramethylrhodamine methyl ester (TMRM) and tetramethylrhodamine ethyl ester (TMRE) [83]. In unstimulated platelets with intact Δψm, TMRM or TMRE diffuses into the mitochondrial matrix, where it is sequestered temporarily resulting in a high fluorescent readout. Upon platelet activation, subsequent Δψm loss causes TMRM or TMRE to translocate out of the mitochondrial matrix and cells, thereby, decreasing fluorescent signal [41]. These cationic dyes can be used in flow cytometry or fluorescence microscopy to detect changes in Δψm in living platelets. Although Δψm loss occurs in both procoagulant and apoptotic platelets, the loss in Δψm differs in timing. While it takes time for apoptotic platelets to experience Δψm loss, procoagulant platelets undergo depolarization quickly [84]. Thus, this marker should be used in conjunction with other markers to correctly identify procoagulant platelets. Instead of assessing α_IIb_β_3_ integrin affinity as a marker for procoagulant platelet identification, Δψm loss can be used in veterinary species given the lack of suitable antibody for detection of activated integrin [46]. Given the sensitive nature of feline platelets and the rapid loss of Δψm following strong agonists stimulation, we devised a stepwise approach to first characterize Δψm by TMRM loading prior to PS and P-selectin detection to avoid in vitro activation. This also allows for a more physiologic assessment as changes in Δψm can be detected first prior to the addition of exogenous calcium for annexin V analysis.

#### 6.5.2. 4-[N-(S-Glutathionylacetyl)amino]phenylarsonous Acid (GSAO)

GSAO is a cell death marker that can be used to differentiate procoagulant platelets from apoptotic platelets when it is used in conjunction with P-selectin [45,78,85]. GSAO enters necrotic or procoagulant platelets via an organic anion-transporting polypeptide and remains within the cytoplasm by forming covalent bonds with protein dithiols. Its use in conjunction with P-selectin to detect apoptotic and necrotic platelets has been evaluated in vitro in washed human platelets [45]. Necrotic and procoagulant platelets share a similar pathway in which both cellular processes are dependent on CypD. When human platelets were activated with thrombin in the presence or absence of collagen, inhibition of CypD by cyclosporine A significantly decreased GSAO detection in platelets. This demonstrates that GSAO may identify procoagulant platelets that are CypD-dependent. When GSAO was injected in mice subjected to arterial injuries by ferric chloride, GSAO was found to be colocalized within platelets and fibrin strands of the occlusive thrombi, suggesting that GSAO may be capable of identifying procoagulant platelets in vivo [45]. Despite the advantages of using GSAO and P-selectin to accurately differentiate procoagulant and apoptotic platelets, GSAO is not yet commercially accessible.

#### 6.5.3. Fibrinogen Binding

Although the binding affinity of integrin α_IIb_β_3_ to fibrinogen is decreased in procoagulant platelets, a decrease in fibrinogen binding is not an accurate assessment to monitor α_IIb_β_3_ affinity in procoagulant platelets. COAT platelets, which are a subset of procoagulant platelets, have a high affinity to fibrinogen due to the high concentration of coagulation proteins that are localized on the platelet surface [86,87]. However, because increased binding to labeled fibrinogen is a characteristic of COAT platelets, fibrinogen binding can be utilized in conjunction with other markers to detect procoagulant platelets. Washed platelets are ideal given that the fibrinogen in platelet-rich plasma may interfere with the binding of exogenous labeled fibrinogen. The addition of a standardized concentration of plasma protein in the form of platelet poor plasma or pooled plasma is also essential to facilitate fibrinogen binding on procoagulant platelets.

#### 6.5.4. Platelet-Derived Microvesicles

PDMVs are released from budding of membrane surfaces of both apoptotic and procoagulant platelets. While PDMVs are not specific to procoagulant platelets, their elevation in clinical patients often signals a prothrombotic state as they provide an abundance of procoagulant phospholipids to facilitate thrombin generation [54,62,63,64,65]. The shedding of PDMVs from procoagulant platelets can be quantified by using antibodies specific to platelet integrins such as integrin β_3_ (CD61) and/or P-selectin. In addition, PS on PDMVs is commonly detected by fluorophore-conjugated annexin V. Due to the small size of microvesicles, optimization of flow cytometry detection using specialized calibration beads is crucial for accurate and precise quantification [88,89].

### 6.6. Laboratory Detection of Procoagulant Platelets in Veterinary Medicine

Given the species differences in platelet physiology, a modified approach in inducing procoagulant platelet formation and its assessment is needed. In general, platelet morphology across companion animals is similar to human platelets, but platelet size varies greatly. For example, feline platelets are highly variable in size with many large circulating platelets and a mean platelet volume of about 15.1 fL compared to the reported reference range of 7.2–11.7 fL in humans [90,91]. Canine platelets, on the other hand, are moderately variable in size but have similarly sized platelets to humans (~7.2 fL) [92]. Overall platelet size is associated with the functional capacity and hemostatic potential of platelets. Large platelets not only express higher levels of integrins and adhesion molecules but also exhibit higher mitochondrial activities [93]. In addition, there are species differences in the types and expression of glycoproteins on the platelet membrane [94]. This suggests that platelets from different species may have variable responses to the agonist type and concentrations. Due to the differences in hemostatic potential and response to agonists, investigators should tailor their experimental approaches and assays to study procoagulant platelets according to the species of interest.

Because procoagulant platelet formation requires ex vivo or in vitro stimulation, the procedures used to isolate platelets may inadvertently lead to undesirable spontaneous or iatrogenic activation that can interfere with assay interpretations. For instance, unlike humans and pigs, feline and canine platelets are cold sensitive with an activation threshold at around 30 °C [11]. When platelets are processed in ambient temperatures below this activation threshold, membrane phospholipids undergo phase transition and subsequent lipid raft formation, causing increased membrane permeability [95]. Furthermore, feline platelets have higher sensitivity to thrombin and exogenous calcium [71,73,96]. The commonly used concentration of thrombin (1 U/mL) to activate human platelets can result in spontaneous aggregation and clotting in feline platelets [30,78]. Exogenous calcium that is required for annexin V binding should be added in aliquots to avoid spontaneous aggregation that can hinder flow cytometry analysis.

Due to the aforementioned species differences in platelet physiology, the ISTH guidelines may not be fully applicable to veterinary species. To overcome this, the authors have devised a stepwise approach to characterize procoagulant platelet formation using flow cytometry in cats [46]. Given the lack of specific antibodies to detect high-affinity α_IIb_β_3_ integrins in veterinary species, other markers such as fibrinogen binding and Δψm along with PS and P-selectin have been used to detect procoagulant platelets to differentiate between procoagulant and apoptotic platelets in animals [46,66]. In our modified approach to assess procoagulant platelet markers sequentially in feline platelets, we showed that Δψm can be assessed first to avoid mitochondrial injuries and reactive oxygen species generation due to the isolation process and prolonged handling. Subsequently, PS externalization and P-selectin can be assessed with additional calcium to facilitate supramaximal intracellular calcium. However, due to the requirement of exogenous calcium for annexin V binding, washed platelets without plasma proteins should be utilized to minimize fibrin formation that can obscure P-selectin detection. In dogs, the authors have successfully detected simultaneous binding of conjugated fibrinogen and P-selectin on procoagulant platelets.

**Figure 3:**
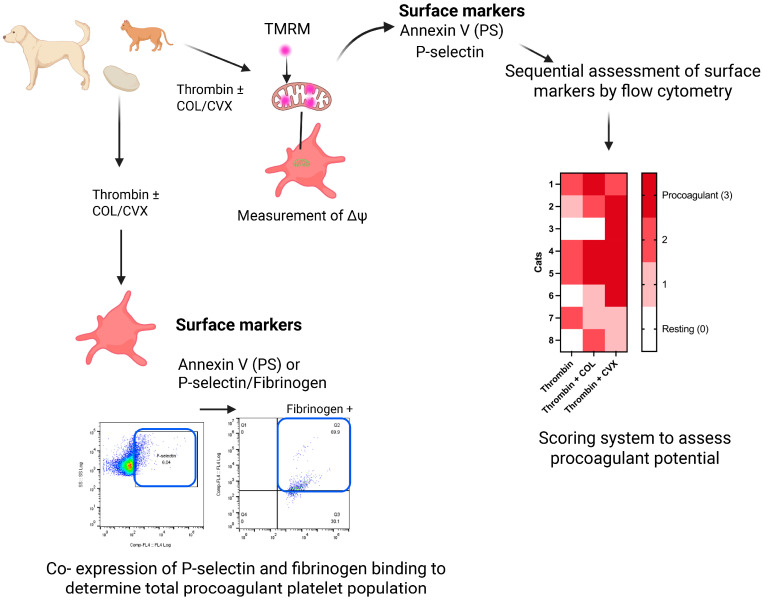
**Representative flow cytometry workflow for detection of procoagulant platelets in companion animals.** Stimulation of isolated platelets from dogs and cats with thrombin or in combination with collagen (COL) or convulxin (CVX). Platelets were then assessed for procoagulant markers by flow cytometry, including mitochondrial membrane potential by TMRM staining, phosphatidylserine exposure by annexin V, P-selectin expression, and fibrinogen binding. In dogs, procoagulant platelets can be identified based on co-expression of P-selectin, PS, and fibrinogen binding. Flow cytometry plots illustrate the gating strategy for identifying P-selectin (blue box) and fibrinogen positive (blue box) platelets. In cats, procoagulant platelets can be measured by sequential analysis of TMRM, annexin V, and P-selectin. A scoring system like the one demonstrated here can be used in cats to evaluate procoagulant potential based on marker expression across stimulation conditions. A representative heatmap summarizes the procoagulant scores across individual animals and experimental conditions.

Given the species differences in platelet response to agonists, investigators are encouraged to conduct dose response testing. In cats and dogs, the authors have been successful in inducing procoagulant phenotypes by stimulating platelets with thrombin in the presence of GPVI agonists, collagen, and convulxin. When simultaneous detection of surface markers is not possible, a scoring system, similar to the one we devised, can be used to evaluate the procoagulant tendency of platelets in individual animals. Response of each marker was scored according to an arbitrary cut-off established in platelets treated with thrombin and collagen or convulxin on flow cytometry. The scores for each marker were then tabulated to yield a total procoagulant tendency score in each individual animal (Figure 3). Overall, thrombin and convulxin were the most potent combination of agonists in inducing a procoagulant phenotype followed by collagen in feline platelets [46]. In dogs, washed gel-filtered platelets were standardized in calcium containing buffer with 10% canine plasma and conjugated human fibrinogen. Platelets were then treated with activators including thrombin, GPVI agonists, and neutrophil supernatant for a short period of time before codetection of P-selectin and fibrinogen binding (Figure 3) [70].

## 7. Novel Anticoagulant Therapies: Targeting Procoagulant Platelets

Existing anti-platelet drugs target aggregatory platelets by limiting the initial platelet plug formation and decreasing platelet-to-platelet interactions indirectly or directly [4]. A limitation of this approach, however, is the inadequate adhesion to thrombogenic surfaces and thrombus formation predisposing patients to life-threatening iatrogenic hemorrhages [4]. On the other hand, monotherapy with anti-platelet drugs may be insufficient to prevent different types of pathologic thrombosis given the synergistic connections between primary and secondary hemostasis in platelet function and thrombin generation [97]. In fact, cats with cardiogenic arterial thromboembolism experience recurrent thrombotic events despite receiving antithrombotic treatments, with a reported one-year recurrence rate ranging from 25% to 61% [27]. For this reason, procoagulant platelets can be a potential therapeutic target to fine tune the hemostatic response of platelets by limiting thrombin generation. However, a potential challenge of procoagulant platelet inhibition is the increased risk of bleeding diatheses such as those reported in humans and dogs with Scott syndrome. Although there are no licensed inhibitors that specifically target procoagulant platelet formation, multiple studies have shown promising results of potential therapies directed at procoagulant platelets.

### 7.1. Carbonic Anhydrase Inhibitors

**Figure 4:**
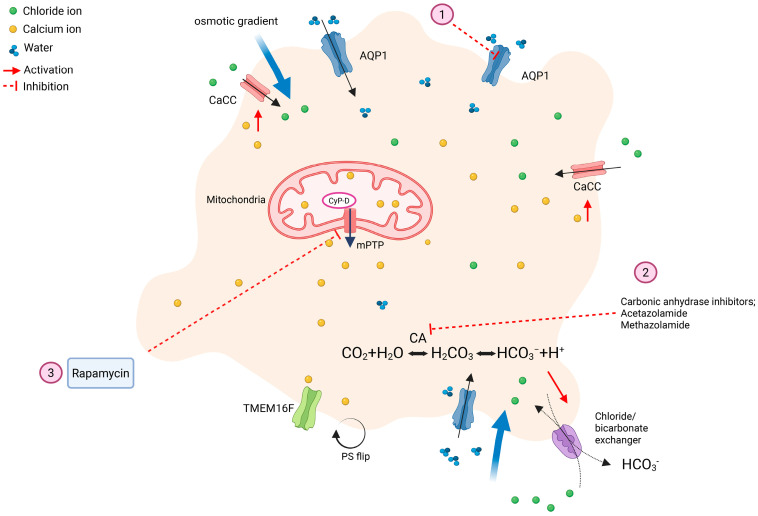
Potential therapeutic targets to block procoagulant platelet formation. Binding of strong agonists such as thrombin and collagen to their receptors initiates a downstream cascade of events leading to an initial increase in intracellular calcium. This calcium elevation activates CaCC, which promotes water entry via AQP1 by osmotic gradient generated by increased chloride ions. (**1**) AQP1 inhibition diminishes platelet spreading and thrombus formation. Chloride ions also enter the cytoplasm through chloride/bicarbonate antiporter. Export of bicarbonate is facilitated via carbonic anhydrase, which catalyzes the conversion of carbon dioxide (CO_2_) and water (H_2_O) to carbonic acid, bicarbonate, and protons. (**2**) CA inhibitors such as acetazolamide and methazolamide block CA to modulate procoagulant platelet generation. (**3**) Rapamycin may reduce procoagulant platelet generation by preserving the mitochondria membrane potential.

Because chloride influx is dependent on the chloride/bicarbonate exchange via CA, CA inhibitors may be a viable option to inhibit procoagulant platelet formation (Figure 4). CA inhibitors like acetazolamide (ACZ) and methazolamide (MTZ) have been shown to modulate procoagulant platelet generation by blocking CA while sparing platelet aggregation and secretion in vitro [98,99,100]. Moreover, an in vivo study showed that mice with arterial thrombosis and deep vein thrombosis also indicated the ability of ACZ and MTZ to diminish thrombosis [43,101]. Studies by Agbani et al. indicated that ACZ diminished membrane ballooning of human platelets in vitro along with decreasing thrombus generation in an in vivo mouse model [43,98]. These CA inhibitors possibly modulate the chloride ions entry required for membrane ballooning.

Given that CA is widely expressed throughout the body, clinically available CA inhibitors are largely non-selective [102]. This is complicated by adequate dosing to ensure cellular penetration while keeping the adverse effects minimal. The clinical benefits of available CA inhibitors as an adjunct or stand-alone anti-platelet therapy require further evaluations. In addition, their combination with another anti-platelet drug is also not suitable for all patient groups given the lack of selectivity. However, some patients can still benefit from their inhibitory effect on procoagulant platelets, including patients after myocardial infarction surgery. Minimalization of procoagulant platelet activity while sparing aggregation and secretion can assist the tissue healing process in those patients. Further research is required for more feasible and selective CA inhibitors that target procoagulant platelets.

### 7.2. Aquaporin-1

As explained above, specific AQPs are essential for water entry down the osmotic gradient established by sodium and chloride influx to induce ballooning and spreading of procoagulant platelets (Figure 4). There are, however, challenges in developing inhibitors against AQPs, since there are 13 known isoforms of AQPs in humans. While they do not have identical amino acid sequences, they share similarities in their overall protein structures, especially the conserved pore-forming regions. Because AQPs are wildly expressed in different tissues throughout the body, creating a specific AQP inhibitor could be a challenge. Limited research exists regarding the subtypes of AQPs expressed in platelets from different species. RNA sequencing in human platelets demonstrated the expression of AQPs 1, 3, 4, 9, and 11 [103]. Further research is required to identify the AQP isoforms in platelets along with their localization and patterns of expression in veterinary species.

### 7.3. Rapamycin

Rapamycin is an inhibitor of mammalian/mechanistic target of rapamycin protein complex-1 (mTORC1), which increases cellular autophagy (Figure 4). Rapamycin also asserts its inhibitory effect by binding to the FK506-binding protein-12 (FKBP12), creating a complex that leads to mTORC1 inhibition [104]. Because mTORC1 is expressed in almost all cell types, rapamycin has many pharmacological effects and potential benefits like immune modulation and tissue remodeling [104]. The effects of rapamycin on platelets have largely been limited to in vitro studies with conflicting results in regard to platelet adhesion, aggregation, and secretion. While in some studies rapamycin increased platelet aggregation and secretion in response to ADP and thrombin, another study showed that rapamycin decreases collagen-induced platelet aggregation and spreading [105,106,107]. Another group showed that rapamycin had no significant effect on platelet aggregation upon stimulation with SFLLRN, a PAR1 agonist [108]. When it comes to procoagulant platelets, there is limited evidence on the effects of rapamycin on specific procoagulant phenotypes. One in vitro study in human platelets showed that rapamycin was effective at reducing procoagulant platelet generation by maintaining ΔΨm, PS exposure, thrombin generation, and ballooning of platelets by a mTORC1-independent mechanism. The investigators found that while pre-treatment of platelets with mTOR inhibitors like KU0063794 and WYE687 did not affect PS externalization and platelet ballooning, treatment with FK506 (tacrolimus), which forms a complex with FKBP12 without modulating mTORC1 activity, prevented the loss of ΔΨm and exposure of the PS to the same extent as rapamycin. Together, these data indicate that the mechanism of action of rapamycin on the procoagulant platelets is separate from its inhibitory effect on mTORC1 [109].

Our recent ex vivo study in cats demonstrated that intermittent dosing of rapamycin orally every 7 days for 4 consecutive weeks was well-tolerated and had notable modulating effects on platelet activation and procoagulant platelet formation. Specifically, rapamycin mitigated the loss of ΔΨm at 3, 24, and 48 h after the last rapamycin dose upon platelet treatments with thrombin in the presence of collagen or convulxin. PS externalization was also reduced at 24 h upon treatment of platelets with thrombin and collagen. This is the first ex vivo study demonstrating the efficacy of rapamycin in reducing procoagulant platelet potential in a large animal model [110]. While a long term pharmacodynamic study is needed in clinical feline patients, the protective and modulating effects of rapamycin on the platelet mitochondria and procoagulant platelets coupled with its ameliorating effects studies in humans, mice, and cats with subclinical HCM showing that rapamycin can safely reduce the progression of left ventricular hypertrophy and improve overall cardiac output demonstrates rapamycin’s potential to be used clinically in the management of HCM [109,111,112,113,114]. Further studies are needed to evaluate the potential synergistic effects of rapamycin and anti-platelet drugs on platelet function and prevention of pathogenic thrombosis in animals and humans.

## 8. Conclusions

Procoagulant platelets are important components of a thrombus, providing increased surface areas for coagulation complexes, fibrinogen binding, and thrombin generation. However, increased platelet heterogeneity in certain diseases may favor the formation of procoagulant platelet formation, increasing thrombotic risks in humans and animals. Despite their potential contribution to pathogenic thrombosis, clinical evaluation of procoagulant platelets remains a challenge given the species variation in platelet physiology and unstandardized approach in their laboratory evaluations. Nevertheless, future research should focus not only on the role of procoagulant platelets in pathogenic thrombosis but should also identify specific therapeutic targets to accompany existing anti-platelet therapies. Research over the years has shed light on their distinct features and physiology, which can serve as potential therapeutic targets without affecting the normal aggregatory function of platelets. The potential drugs that were identified, such as AQP and CA inhibitors, along with rapamycin, which can offer synergistic antithrombotic effects when used in combination with anti-platelet drugs, could further decrease thrombosis risk by targeting multiple pathways in platelets. Additional research is required before proceeding with clinical trials for these drugs. While there are multiple assays available to study procoagulant platelets in laboratory settings, there is no specific assay that can be used in clinics. A clinical assay evaluating circulating procoagulant platelets levels could be beneficial for identifying at risk patients who could be candidates for additional anti-platelet therapy. Although most of our understanding of procoagulant platelets arises from human and murine studies, veterinary research has made some advancements in studying procoagulant platelets in large animal models which hold translational potential.

## Figures and Tables

**Table 1:** Summary of features and characteristics of aggregatory, procoagulant, and apoptotic platelets.

	Aggregatory Platelets	Procoagulant Platelets	Apoptotic Platelets
Role in coagulation	Platelet adhesion, aggregation, and clot retraction	Providing a procoagulant membrane surface for clotting factor complex formation thrombin generation	Programmed cell death regulating platelet lifespan
αIIbβ3 integrin	In high affinity of state or “active”	Integrin in low-affinity state or “inactive” Likely decreased expression	Integrin increases thrombin-mediated platelet apoptosis
Morphology	Develop numerous filopodia and lamellipodia resulting in the subsequent spreading to increase surface areas	Balloon-shaped without pseudopodia along with their PS exposed	Formation of apoptotic bodies, reduction in cell size with membrane blebbing
Cytosolic calcium levels	Oscillatory cytosolic calcium concentrations	Sustained intracellular hypercalcemia through SOCE	Calcium independent
Agonist	Formed upon stimulation of various agonists such as ADP, thromboxane A_2_, thrombin, collagen, epinephrine and serotonin	Formed upon stimulation of strong agonists, notably co-stimulation of thrombin and collagen/convulxin	High concentrations of thrombin induce Bak/Bax activation via PAR1 in a caspase dependent process
ΔΨ_m_	Negative on matrix side (hyperpolarized)	Depolarized	Depolarized
MPTP formation	Not required	Required	Required

**Table 2:** Summary of platelet markers to evaluate procoagulant platelets.

Marker	Possible Detection Method(s)	Procoagulant Platelets	Apoptotic Platelets	Aggregatory Platelets	Comments
PS	Flow cytometry: Annexin V or Lactadherin	Yes	Yes	No	Must be used in conjunction with other markers to distinguish procoagulant platelets from apoptotic ones Requires exogenous calcium
P-selectin	Flow cytometry: CD62P conjugated antibodies	Yes	No	Yes	Used in combination with other markers to distinguish between apoptotic and procoagulant platelets
Δψm	Flow cytometry Fluorescence microscopy	Yes	Yes	No	Platelets must be alive and analyzed within a limited time frame Must be used in conjunction with P-selectin to verify procoagulant platelets
Activated αIIbβ3 integrin	Flow cytometry: (PAC-1, JON/A) Western blot	No	No	Yes	Surface markers are specific to human (PAC-1) and murine platelets (JON/A) only No specific markers available in other species
GSAO	Flow cytometry Fluorescence microscopy	Yes	No	No	Co-expression of P-selectin and GSAO can differentiate between procoagulant and apoptotic platelets GSAO is not commercially available yet
Fibrinogen binding	Flow cytometry Confocal microscopy Light transmission aggregometry	Yes	No	Yes	Fibrinogen is retained with high affinity on the procoagulant platelet surface, thus, not ideal to be used as an assay for detection of activated integrin
Platelet-derive (PDMVs)	Flow cytometry	Yes	Yes	No	Protocol must be standardized using calibration beads and optimized to flow cytometers Technically challenging
Cytosolic calcium	Fluorescent dyes using microplate readers	Yes (sustained)	No	Yes (transient)	Assay kits must be sensitive enough to detect changes in [Ca_i_] Only procoagulant platelets experience sustained increase in [Ca_i_] Assays can be technically challenging
